# Intravenous Thrombolysis Administration 3–4.5 h After Acute Ischemic Stroke: A Retrospective, Multicenter Study

**DOI:** 10.3389/fneur.2019.01038

**Published:** 2019-10-15

**Authors:** Yu-Wei Chen, Sheng-Feng Sung, Chih-Hung Chen, Sung-Chun Tang, Li-Kai Tsai, Huey-Juan Lin, Hung-Yu Huang, Helen L. Po, Yu Sun, Po-Lin Chen, Lung Chan, Cheng-Yu Wei, Jiunn-Tay Lee, Cheng-Yang Hsieh, Yung-Yang Lin, Shoou-Jeng Yeh, Li-Ming Lien, Jiann-Shing Jeng

**Affiliations:** ^1^Department of Neurology, Landseed International Hospital, Taoyuan, Taiwan; ^2^Stroke Center and Department of Neurology, National Taiwan University Hospital, Taipei, Taiwan; ^3^Division of Neurology, Department of Internal Medicine, Ditmanson Medical Foundation Chiayi Christian Hospital, Chiayi, Taiwan; ^4^Department of Neurology, National Cheng Kung University Hospital, Tainan, Taiwan; ^5^Department of Neurology, Chi Mei Medical Center, Tainan, Taiwan; ^6^Department of Neurology, China Medical University Hospital, Taichung, Taiwan; ^7^Department of Neurology, Mackay Memorial Hospital, Taipei, Taiwan; ^8^Department of Neurology, En Chu Kong Hospital, New Taipei City, Taiwan; ^9^Department of Neurology, Taichung Veterans General Hospital, Taichung, Taiwan; ^10^Department of Neurology and Stroke Center, Taipei Medical University–Shuang Ho Hospital, New Taipei City, Taiwan; ^11^Taipei Neuroscience Institute, Taipei Medical University, New Taipei City, Taiwan; ^12^Department of Neurology, School of Medicine, College of Medicine, Taipei Medical University, Taipei, Taiwan; ^13^Department of Neurology, Chang Bing Show Chwan Memorial Hospital, Changhwa, Taiwan; ^14^Department of Neurology, Tri Service General Hospital, Taipei, Taiwan; ^15^Department of Neurology, Tainan Sin Lau Hospital, Tainan, Taiwan; ^16^Department of Neurology and Department of Critical Care Medicine, Taipei Veterans General Hospital, Taipei, Taiwan; ^17^Department of Neurology, Cheng Ching General Hospital, Taichung, Taiwan; ^18^Department of Neurology, Shin Kong WHS Memorial Hospital, Taipei, Taiwan

**Keywords:** acute ischemic stroke, tissue plasminogen activator, intravenous thrombolysis, 3–4.5 h after stroke onset, functional recovery

## Abstract

**Background and Objectives:** Intravenous recombinant tissue plasminogen activator (rt-PA) has been approved for acute ischemic stroke (AIS) within 3 h after onset and the treatment was then extended to 4.5 h. However, the Food and Drug Administration did not approve the indication in the expanded time window. This retrospective, matched cohort study aims to investigate the effectiveness and safety of rt-PA in AIS at 3–4.5 h after onset.

**Materials and Methods:** The treatment group included AIS patients receiving rt-PA at 3–4.5 h after onset, otherwise complying with the regulation, in the stroke registries in 16 hospitals between 2008 and 2017. The control group included age- and sex-matched patients not receiving intravenous thrombolysis from the same registries, excluding those with contraindications. The primary outcome was modified Rankin Scale (mRS) 0–1 at day 90. The safety outcomes were any intracerebral hemorrhage (ICH), early neurological deterioration and 3-month mortality.

**Results:** Each group had 374 patients. There were 34.0% of patients with 3-month mRS 0-1 in the treatment group vs. 22.7% in the control group with an odds ratio of 1.75 (95% confidence intervals, 1.27 to 2.42, *P* = 0.001). There was no difference in symptomatic ICH, early neurological deterioration and 3-month mortality rates between two groups. The 3-month mRS and symptomatic ICH did not differ significantly in patients receiving standard dose or low dose of rt-PA.

**Conclusions:** Our results support the prescription of rt-PA in AIS patients 3–4.5 h after onset as an effective and tolerable treatment in their functional recovery.

## Introduction

Stroke is the second most common cause of death, accounting for 11% of total deaths (~ 6.3 million deaths) in 2015 (1). More than 80% of strokes are ischemic strokes (2), and the emergency management of patients with acute ischemic stroke (AIS) has been emphasized to improve the prognosis (3). After the efficacy of intravenous recombinant tissue plasminogen activator (rt-PA) was proved in 1995 (4), it was first approved for AIS treatment within 3 h of stroke onset in the United States of America (USA) in 1996 and has been then approved in the European Union and other countries subsequently, including Taiwan. In 2008, the European Cooperative Acute Stroke Study (ECASS) III demonstrated an increased likelihood of having a favorable outcome in patients treated with rt-PA within 3–4.5 h of the stroke onset compared to the placebo group, along with an increased risk of symptomatic intracranial hemorrhage (5). The third International Stroke Trial (IST-3) found that rt-PA treatment within 6 h of AIS could improve the functional outcome of patients assessed and did not increase the incidence of death at 6- and 18-month follow-ups (6, 7). Studies in China and Japan comparing the outcome and safety between patients using rt-PA within 3 and 3–4.5 h of stroke onset showed no difference in functional outcome, rates of symptomatic intracerebral hemorrhage (SICH) and mortality rates in two time window groups (8, 9).

To date, guidelines for the early management of AIS patients from professional organizations in the USA, Europe, Japan, China, and Taiwan strongly recommend the extension of the intervention time span to 4.5 h after the onset of AIS (3, 10–13). Although evidence from randomized control trials and related meta-analysis supports the efficacy of rt-PA within 3–4.5 h of AIS onset, little information is available from the studies in Asian populations in the literature. The Food and Drug Administration (FDA) in the USA and in Taiwan have not approved the indication of rt-PA at 3–4.5 h after AIS onset. Therefore, we planned a multicenter, retrospective, matched cohort study in Taiwan to collect clinical real-world data aiming to evaluate the effectiveness and safety for AIS patients receiving rt-PA between 3 and 4.5 h after onset.

## Materials and Methods

### Study Design

The study was a multicenter, retrospective, matched cohort study in Taiwan to investigate the effectiveness and safety of patients who had experienced AIS with or without the administration of rt-PA therapy at 3–4.5 h after the stroke onset. Patients with other additional reperfusion or interventional therapy were excluded, and data were extracted from databases of 16 participating hospitals in Taiwan Stroke Registry (TSR). The TSR is a multicenter, pre-specified prospective registry, recruiting patients from more than 50 centers in Taiwan (14). This current study planned to enroll at least 500 eligible patients in the treatment and control groups at a ratio of 1:1 according to whether they received rt-PA therapy or not in the emergency room (ER). In general, information on the basic characteristics of eligible patients (e.g., demographics and medical history), the dates of admission and discharge, the results of laboratory examinations, clinical medications and evaluation for the stroke, and the follow-up outcome were prospectively collected. The Full Analysis Set (FAS) population was utilized for all the analyses in each group to evaluate functional outcomes assessed by a modified Rankin Scale (mRS), the neurological status, mortality, and the incidence of SICH. The TSR was approved by Institutional Review Board of each participating hospital, and written informed consent for participation in the registry program was obtained from each patient or his/her legal representatives with the approval of attending physicians. A numeric code was assigned to each patient whose clinical data were in the registry and the database contained no individual identification numbers or any privacy data. The whole data set as a large database has been approved for big data analytics for research topics, such as the one in this study.

### Study Population

The inclusion criteria were based on the local license and reimbursement regulation in Taiwan, except the rt-PA treatment windows of 3–4.5 h for otherwise eligible patients. The treatment and control groups included patients with an age ≥18 years and were diagnosed with an AIS. The diagnosis was based on clinical evaluation and was documented by appropriate diagnostic procedures of brain computed tomography and/or magnetic resonance imaging, confirmed by in-charge neurologists, and radiologists. AIS were further classified into 5 subtypes by the Trial of Org 10172 in the Acute Stroke Treatment (TOAST) classification (15), including large artery atherosclerosis, small artery lacunes, cardioembolism, other specific etiology and undetermined etiology. Stroke of undetermined etiology includes those with negative or incomplete study, and two or more identified causes (15). AIS patients with pre-morbid functional status of mRS >1 and thrombolyzed patients with final diagnosis other than AIS (stroke mimics) were excluded. The presenting scores of NIH Stroke Scale (NIHSS) of both groups were between 4 and 25, and none of the patients received any other reperfusion therapy (i.e., intra-arterial thrombolysis or endovascular thrombectomy). Patients in both groups did not participate in any clinical trials regarding the treatment of AIS.

In the treatment group, patients received rt-PA within 3–4.5 h after stroke onset, otherwise in accordance with the regulations of Taiwan FDA and of the reimbursement of the National Health Insurance in Taiwan (16). In the control group, age- and sex-matched patients arriving at the ER within 2–4.5 h after stroke onset, but not receiving rt-PA, at the same hospital if possible, were included. The lower limit of 2 h onset-to-door time was defined because they were probably not able to receive rt-PA treatment within 3 h. Patients in the control group were without any obvious contraindication to rt-PA treatment, including an abnormal hemogram profile of an international normalized ratio ≥1.7 and platelet counts <10^5^/μL, experiencing a previous hemorrhagic stroke, or were not receiving rt-PA treatment based on the investigator's discretion.

The standard dose of 0.9 mg/Kg of rt-PA in AIS has been approved by FDA in Taiwan and it was regularly used in the daily practice. In 2016, the contraindication of rt-PA for AIS patients older than 80 years was removed in the label, with adding the warning that rt-PA should be cautiously used in patients older than 80 years. The label also provided that low dose of intravenous rt-PA was associated with lower risk of SICH in Low-Dose versus Standard-Dose Intravenous Alteplase in Acute Ischemic Stroke (ENCHANTED) study (17) and Taiwan Thrombolytic Therapy for Acute Ischemic Stroke (TTT-AIS) study (18). The TTT-AIS study also demonstrated that patients older than 70 years treated by standard dose were less likely to have 3-month mRS 0–2 than those treated by lower dose. We had a subgroup analysis for the baseline characteristics and outcomes of patients receiving standard dose (0.9 mg/Kg) and low dose (0.6 mg/Kg) in the treatment group.

### Outcomes

The primary outcome was the percentage of patients with modified Rankin Scale (mRS) scores of 0–1 at 90 days after AIS. The secondary outcomes were: (1) the percentage of patients with mRS scores of 0–2 at 90 days after AIS; (2) the percentage of patients with early neurological deterioration, defined as deterioration with NIHSS scores ≧2 within 72 h after AIS (19, 20), and (3) the mortality rates within 90 days after the onset of AIS. The safety assessments included the percentages of patients with any intracerebral hemorrhage within 36 h after the administration of rt-PA, and of patients with SICH determined by three well-accepted definitions. Safe Implementation of Thrombolysis in Stroke-Monitoring study (SITS-MOST) was defined as a local or remote parenchymal hematoma type 2 on the imaging scan obtained 22 to 36 h after treatment, plus neurologic deterioration, as indicated by a score on the NIHSS that was higher by 4 or more points than the baseline value or the lowest value between baseline and 24 h, or hemorrhage leading to death (21). European Cooperative Acute Stroke Study III (ECASS III) was defined as any hemorrhage with neurologic deterioration, as indicated by an NIHSS score that was higher by 4 points or more than the value at baseline or the lowest value in the first 7 days, or any hemorrhage leading to death. In addition, the hemorrhage must have been identified as the predominant cause of the neurologic deterioration (5). National Institute of Neurological Disorders and Stroke (NINDS) was defined as an intracranial hematoma not been seen on a previous CT scan but was subsequently either a suspicious of hemorrhage or any decline in neurologic status (4). To detect intracranial hemorrhage, brain CT or MRI scans were required at 24 h or 7 to 10 days after the onset of stroke when clinical findings suggested hemorrhage.

### Data Quality Assurance

To ensure data accuracy, completeness, and reliability, the following procedures were enforced. Before collecting data for the study, the principal investigator (J. S. Jeng) conducted a Site Initiation Visit meeting to instruct the investigators and study coordinators on the protocol, the guideline for the completion of the Case Report Form (CRF), and for procedures of the study. The clinical data of patients in the participating hospitals were recorded based on the instructional materials, were audited regularly and were reported to the Data Monitoring Board. A visual review and standard computer editing monitored the completeness and accuracy of data on the CRFs.

To protect the safety of the subjects and to ensure data accuracy, completeness, and reliability, the research staff preserved documented data from all sources on CRFs, including lab test results, chart records, treatment conditions, physical examination, concomitant medications, and any adverse events.

### Statistical Analysis

A sample size of at least 250 patients in each of the treatment and control groups was required to provide an α error and power of 0.05 and 80%, respectively.

All data extracted from the eligible patients were evaluated. No withdrawal criteria were applied for the retrospective study. Eligible patients not receiving rt-PA therapy were categorized into the control group and patients treated with rt-PA into the treatment group at a ratio of 1:1. The demographic and clinical characteristics between groups were compared by Student's *t*-test or chi-square test. Since this study was a retrospective study, the study did not interfere with the patients' medical procedure and complied with routine practice in Taiwan.

As a retrospective study, the FAS population consisting of all eligible patients used for the assessment of effectiveness and safety. We assessed mRS scores and mortality rates of patients by the chi-square test, or other non-parametric methods (Mann-Whitney *U*-test). The variables significantly associated to primary outcome with mRS 0–1 at 90 days in the univariate analysis were further analyzed by a logistic regression model. Safety outcomes of the percentages of patients with intracerebral hemorrhage (ICH) were assessed by a chi-square test or other non-parametric methods (Mann-Whitney *U*-test). The mRS shift analysis by Mann-Whitney *U*-test was performed between the treatment and control groups and between the standard dose and low dose sub-groups in the treatment group.

## Results

From January 2008 to December 2017, a total of 748 eligible patients, of whom 502 (67.1%) were males, treated with/without rt-PA (in the treatment and control groups, respectively) within 3–4.5 h after the onset of an AIS were enrolled in our study from 16 participating hospitals at the ratio of 1:1, with 374 patients in each group. All institutes in the study were either medical centers or qualified stroke centers and contributed patients in both treatment and control groups. The characteristics of patients with AIS in the treatment and control groups at baseline are presented in Table 1. The mean age of patients in the treatment and control groups was 66.1 ± 13.2 and 67.8 ± 12.5 years (*P* = 0.290), respectively. The mean NIHSS score was insignificantly higher in the treatment group than in the control group (12.2 ± 6.0 vs. 10.7 ± 6.4, *P* = 0.197). Regarding other variables, including laboratory data and medical histories, there was no statistically significant difference between the two groups (all *P* > 0.05).

**Table 1:** Summary of the demographics of the treatment and control groups.

**Characteristics**	**Treatment (*N* = 374)**	**Control (*N* = 374)**	***P*-value**
Age (year)	66.1 ± 13.2	67.8 ± 12.5	0.290
Male, *n* (%)	251 (67.1)	251 (67.1)	1.000^†^
Body weight (Kg)	65.1 ± 12.7	65.4 ± 12.9	0.669
NIHSS	10 (7–17)	9 (5–15)	0.001
**Laboratory data**			
Systolic BP (mmHg)	161.0 ± 30.7	161.0 ± 32.5	0.434
Diastolic BP (mmHg)	91.6 ± 19.3	90.7 ± 19.2	0.680
Glucose (mg/dL)	154.1 ± 69.8	158.5 ± 75.4	0.100
INR	1.01 ± 0.10	1.01 ± 0.10	0.987
Creatinine (mg/dL)	1.26 ± 1.09	1.35 ± 1.36	0.090
Platelet count (10^5^/mm^3^)	216.8 ± 72.0	212.8 ± 70.1	0.830
**Medical history**, ***N*** **(%)**			
Hypertension	282 (75.4)	291 (77.8)	0.490
Diabetes mellitus	137 (36.6)	160 (42.8)	0.100
Hyperlipidemia	199 (53.2)	201 (53.7)	0.942
Hypercholesterolemia	174 (46.5)	175 (46.8)	1.000
Hypertriglyceridemia	67 (17.9)	64 (17.1)	0.847
Cardiac disease	165 (44.1)	143 (38.2)	0.119
Atrial fibrillation	127 (34.0)	113 (30.2)	0.309
Ischemic heart disease	45 (12.0)	38 (10.2)	0.485
Valvular heart disease	15 (4.0)	10 (2.7)	0.416
Heart failure	30 (8.0)	25 (6.7)	0.488
**Smoking**			
Current smoker	103 (27.5)	98 (26.2)	0.742
Ex-smoker	26 (7.0)	41 (11.0)	0.072
Previous ischemic stroke	75 (20.1)	94 (25.1)	0.115
Previous TIA	11 (2.9)	5 (1.3)	0.205
History of malignancy	21 (5.6)	25 (6.7)	0.648

*Values are present as mean ± standard deviation, numbers (%), or median (interquartile range)*.

*P-value by Student t-test or chi-square test*.

† *Significant difference, P-value < 0.05*. *NIHSS, National Institutes of Health stroke scale/score; BP, blood pressure; INR, international normalized ratio; TIA, transient ischemic attack*.

No statistically significant difference was found between two groups regarding stroke subtypes or type of large artery atherosclerosis in the TOAST classification, except that the percentage of undetermined causes of stroke was higher in the treatment group (27.5 vs. 20.1%) (Supplementary Table 1).

A summary of medications taken prior to the onset of strokes was illustrated in Supplementary Table 2. In general, records of prior medications were not significantly different between the treatment and control groups, except higher percentage of prior aspirin use in the control group.

A shorter average stroke onset-to-door time was observed in the treatment group (128.6 ± 42.8 min vs. 196.0 ± 58.8 min in the control group, *P* < 0.001). But the onset-to-needle time in the treatment group (206.9 ± 25.9 min) was longer than the onset-to-door time in the control group (196.0 ± 58.8 min, *P* = 0.001). The averages of dose in mg per patient and of dose per Kg of administered rt-PA in AIS patients were 48.4 ± 13.4 mg and 0.74 ± 0.14 mg/Kg, respectively. Among these patients, 48.7% (*n* = 182) received the standard dose (0.9 mg/Kg) and 51.3% (*n* = 192) received a low dose (0.6 mg/Kg).

**Figure 1:**
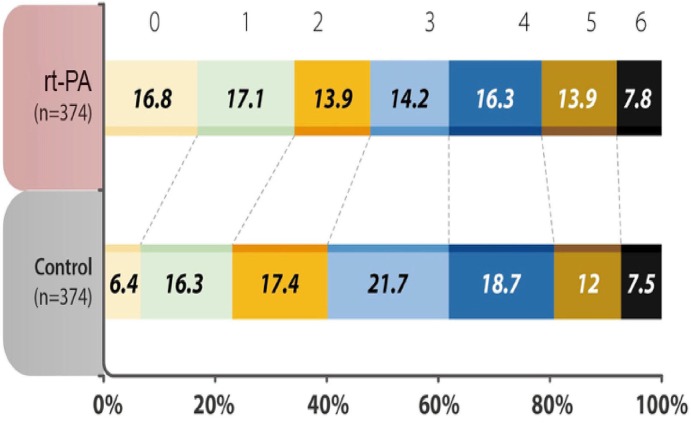
Distribution of acute ischemic stroke patients in each group was evaluated by modified Rankin Scale (mRS) scores at 90 days after the onset of the index stroke. The analysis of primary outcome showed a significantly higher percentages (mRS 0-1) in the recombinant tissue plasminogen activator (rt-PA)-treated group (*P* < 0.001). mRS shift analysis by Mann-Whitney *U*-test showed *P* = 0.04 for 3-month mRS between the treatment and control groups.

The percentage of patients with a mRS score of 0–1 at 90 days after the stroke onset was 34.0% in the treatment group, as compared with 22.7% in the control group, with an odds ratio (OR) of 1.75 (95% Confidence Intervals (CI) 1.27 to 2.42, *P* = 0.001). In terms of the distribution of patients with mRS scores at 90 days after the stroke onset, the shift analysis by Mann-Whitney *U*-test showed *P* = 0.04 between the treatment and control groups (Figure 1).

The proportion of rt-PA-treated patients with a mRS score of 0–2 was 47.9%, which was also significantly higher than patients without rt-PA treatment (40.1%, OR, 1.37, 95% CI, 1.03 to 1.83, *P* = 0.033) (Table 2). Fifteen percent of patients in the treatment group suffered from early neurological deterioration, insignificantly lower than 19.5% in the control group (OR, 0.73, 95% CI 0.61 to 1.06, *P* = 0.101). The incidences of death within 90 days after the onset of AIS were 7.8 and 7.5% in the treatment and control groups, respectively (Table 2).

**Table 2:** Percentage of AIS patients assessed for the primary and secondary endpoints.

**Characteristic**	**Treatment *N* = 374, *n* (%)**	**Control *N* = 374, *n* (%)**	**OR (95% CI)**	***P*-value**
**Primary Endpoint**				
mRS score of 0–1	127 (34.0)	85 (22.7)	1.75 (1.27–2.42)	0.001^†^
**Secondary Endpoints**				
mRS score of 0–2	179 (47.9)	150 (40.1)	1.37 (1.03–1.83)	0.033^†^
Early neurological deterioration	56 (15.0)	73 (19.5)	0.73 (0.61–1.06)	0.101
Incidence of death	29 (7.8)	28 (7.5)	1.04 (0.61–1.78)	0.890

*P-value by chi-square test*.

† *Significant difference, P-value < 0.05*.

*AIS, acute ischemic stroke, mRS, modified Rankin scale, OR, odds ratio, CI, confidence interval*.

Follow-up neuroimaging study by MRI was performed in 195 (52.1%), by CT 170 (45.5%), and by none 9 (2.4%) in the treatment group, as compared with by MRI 237 (63.4%), by CT 105 (28.1%), and by none 32 (8.6%) in the control group. Concerning safety assessment, a significantly more percentages (17.4%) in the treatment group experienced ICH (OR, 2.25, 95% CI 1.43 to 3.53, *P* < 0.001) than 8.6% in the control group. However, the percentage of patients with SICH was not significantly different between the two groups (treatment vs. control: 3.5 vs. 2.4%, 3.5 vs. 2.4% and 5.6 vs. 2.9%, based on three definitions of SICH by SITS-MOST, ECASS III and NINDS) (Table 3).

**Table 3:** Summary of the percentage of patients with ICH after the onset of an ischemic stroke.

**Characteristic**	**Treatment *N* = 374, *n* (%)**	**Control *N* = 374, *n* (%)**	**OR (95% CI)**	***P*-value**
Any ICH	65 (17.4)	32 (8.6)	2.25 (1.43–3.53)	< 0.001^†^
**Symptomatic ICH**				
According to SITS-MOST	13 (3.5)	9 (2.4)	1.46 (0.62–3.46)	0.389
According to ECASS III	13 (3.5)	9 (2.4)	1.46 (0.62–3.46)	0.389
According to NINDS	21 (5.6)	11 (2.9)	1.96 (0.93–4.13)	0.076

*P-value by chi-square test*.

† *Significant difference, P-value < 0.05*.

*OR, odds ratio; CI, confidence interval; ICH, intracerebral hemorrhage; SITS-MOST, Safe Implementation of Thrombolysis in Stroke-Monitoring Study; ECASS III, European Cooperative Acute Stroke Study III; NINDS, National Institute of Neurological Disorders and Stroke*.

The study used a logistic regression model to identify factors affecting the good neurological function of a mRS score of 0–1 at 90 days, and results are shown in Table 4. Being younger, having a lower NIHSS score, receiving rt-PA (adjusted OR 2.38, 95% CI 1.655 to 3.427, *P* < 0.001), and not with any ICH were independent predictors of having better neurological function at 90 days after the onset of an ischemic stroke (*P* < 0.05).

**Table 4:** Multiple logistic regression analysis for the good neurological function (mRS 0-1) at 90 days.

**Characteristic**	**OR (95% CI)**	***P*-value**
Age (years)	0.964 (0.950–0.978)	<0.001^†^
Male sex	1.075 (0.728–1.589)	0.716
NIHSS	0.866 (0.834–0.900)	<0.001^†^
rt-PA use	2.381 (1.655–3.427)	<0.001^†^
Any ICH	0.397 (0.184–0.858)	0.019*

*P-value by multiple logistic regression*.

† *Significant difference, P-value < 0.05*.

*mRS, modified Rankin scale; OR, odds ratio; CI, confidence interval; ICH, intracerebral hemorrhage; NIHSS, National Institutes of Health Stroke Scale/Score; rt-PA, recombinant tissue plasminogen activator*.

### Subgroup Analysis

**Figure 2:**
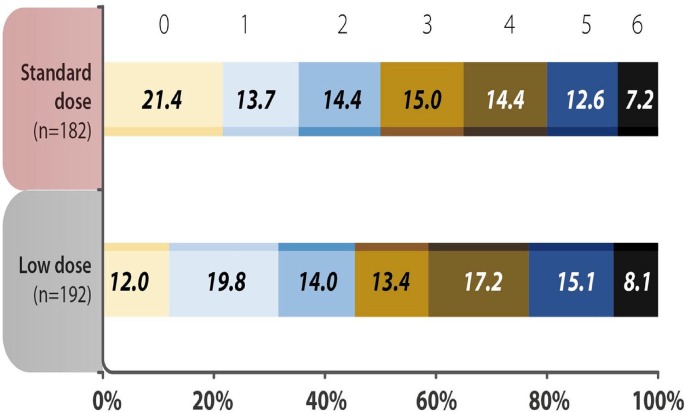
Distribution of acute ischemic stroke patients using different doses of recombinant tissue plasminogen activator (rt-PA) were evaluated by modified Rankin Scale (mRS) scores 90 days after the onset of stroke. The analysis showed no difference in the 3-month mRS scores in the standard vs. low dose rt-PA-treated groups (*P* = 0.346). mRS shift analysis by Mann-Whitney *U*-test showed *P* = 0.12 for 3-month mRS between the standard-dose and low-dose groups.

The baseline characteristics of the standard dose and low dose subgroups were summarized in Supplementary Table 3. Except the patients receiving low dose were older, there were no significant differences between the two groups. The analysis of mRS 0–1 at 90 days, early neurological deterioration, any ICH, SICH and mortality within 90 days showed no difference between subgroups receiving different doses of rt-PA at 3–4.5 h after the stroke onset (Table 5). No variable was considered affecting effectiveness or safety outcomes in patients treated with rt-PA, regardless of whether they received the standard dose or a low dose (all *P* > 0.05) (Figure 2). The mRS shift analysis by Mann-Whitney *U*-test showed *P* = 0.12 between standard dose and low dose groups.

**Table 5:** Logistic regression model analysis for the rt-PA dose.

**Characteristic**	**Standard dose *N* = 182, *n* (%)**	**Low dose *N* = 192, *n* (%)**	**OR (95% CI)**	***P*-value**
mRS score of 0 or 1	66 (36.3)	61 (31.8)	1.222 (0.796–1.876)	0.359
Early neurological deterioration	23 (12.6)	33 (17.2)	0.697 (0.392–1.240)	0.219
Any ICH	29 (15.9)	36 (18.8)	0.821 (0.480–1.406)	0.473
Symptomatic ICH	7 (3.8)	14 (7.3)	0.509 (0.200–1.290)	0.155
Death	13 (7.1)	16 (8.3)	0.846 (0.395–1.812)	0.667

*P-value by logistic regression model*.

*OR, odds ratio; CI, confidence interval; mRS, modified Rankin scale; ICH, intracerebral hemorrhage*.

## Discussion

The study showed effectiveness and safety of rt-PA administered at 3–4.5 h after the onset of AIS when compared with age and sex-matched patients in this multicenter retrospective cohort study. Although evidence from the randomized control trials and meta-analysis support the efficacy of rt-PA in the 3–4.5 h window after AIS, little information is obtained from the investigation in Asian populations in the literature. Our study might be the first case-matched cohort study for the indication in Asian populations. Studies conducted in China and Japan showed comparable outcomes assessed by a mRS score of 0–1 and by mortality rates between the groups of patients using rt-PA within 3 and at 3–4.5 h after the stroke onset. A subgroup analysis of the study comparing the reimbursement (treated <3 h after the onset of AIS) and non-reimbursement (treated 3–4.5 h after the onset) groups for insurance coverage of intravenous rt-PA in Taiwan showed comparable results in functional outcome, in-hospital mortality and SICH (16).

The percentage of rt-PA-treated patients with a 90-day mRS score of 0–1 was 34.0%, significantly higher than 22.7% in the control group. Similar effects were observed in the secondary outcome analysis of a 90-day mRS score of 0–2. Our results are comparable to the results in the meta-analysis including 9 randomized control trials showing 35.3% with a 3-month mRS score of 0–1 in the treatment group in the 3–4.5 h window (22) and to 35.3% (standard dose) and 32.4% (low dose) in a study in Korea (23). However, the value was lower than 52.4% treated in 3–4.5 h window in the ECASS III study (5), which may be explained by that we did not exclude age older than 80 years as in the ECASS III study, reflecting by the older mean age in our study. Besides, there were possible differences between randomized control studies and real-world practice, and among race-ethnicities.

Regarding safety outcomes, the administration of rt-PA had no difference on the risk of SICH by different definitions, early neurological deterioration and the incidence of death at 90 days after the stroke onset, but it was significantly associated with any ICH (17.4%) compared with the control group (8.6%). This observation was consistent with previous studies, in which the incidence rates of ICH were 2.5–27.0% for AIS patients treated with rt-PA beyond 3 h after the onset, higher than patients receiving placebo (all ≤1.0%) (5, 6, 22, 24). Comparable results were also observed in Asian countries with a range in rates of 1.9–12.1%, compared with the placebo group (<1.5%) (8, 25, 26). Extension of infarct volume was the most frequent cause of early neurological deterioration after AIS (27), which could be decreased by thrombolysis (28). A hypothetical analysis showed that the approval of rt-PA treatment within 4.5-h may increase the chance of having a mRS score of 0–1 at 3–6 months (OR 1.37, 95% CI 1.17–1.59) without increasing mortality and SICH rates (29). Although fatal intracranial hemorrhage in 7 days increased in patients treated at 3–4.5 h after the stroke onset in the meta-analysis, adverse effect of the complication was offset by increasing disability-free survival (22).

Intravenous rt-PA may benefit more AIS patients in the extended 3–4.5 h window since only 27% AIS patients were admitted to ER in 3 h (30). The treatment guidelines in the USA, Europe, Japan, and China now all advise to extend the treatment window time to 3–4.5 h after the stroke onset (3, 10–12, 31). Taiwan Stroke Society issued a guideline in 2013 and its update in 2019 to advocate administering rt-PA for AIS patients within 3–4.5 h of the stroke onset (32). However, it still has not been approved by the FDA in the USA and in Taiwan.

Our study supported that patients receiving low dose of rt-PA had similar effectiveness and safety profile as compared with those receiving standard dose in the treatment group in the 3–4.5 h window. The subgroup analysis showed the patients receiving low dose were older than those having standard dose of rt-PA. The practice was supported by the TTT-AIS study in Taiwan demonstrating the lower-than-standard dose administration was associated with less SICH by ECASS III definition and mortality, and with more independence rates in the population older than 70 years (18). Although the randomized control study conducted in Asia didn't support the non-inferiority hypothesis of low dose rt-PA (0.6 mg/Kg) within 4.5 h of AIS (17), the real-world prospective registry in Korea supported the strategy of low dose intravenous thrombolysis (23).

The logistic regression model in our study showed rt-PA treatment is one of the independent factors predicting favorable neurological outcome in patients at 3–4.5 h after AIS onset, as well as younger age, lower NIHSS scores, and absence of ICH. More stroke severity is associated with more risk of SICH (22) and is a strong predictor of functional outcome (5). SICH following thrombolysis was related to early neurological deterioration and poor outcomes (20, 33, 34).

There are limitations of the current study. First, missing data exists since some information were retrospectively collected from the hospital databases. Second, the data were only collected in a short follow-up period after the onset of AIS. Third, it was a non-randomized retrospective study, and we did not apply any population matching methods between the study groups and the characteristics of enrolled patients might be imbalanced. That could have had an influence on the study results. Finally, the regimen of rt-PA was decided at the discretion of different investigators, which might affect the assessment of endpoints. However, the present study demonstrated the effectiveness and safety of rt-PA for patients at 3–4.5 h after the onset of AIS in a real-world setting.

In conclusion, our results support the use of rt-PA for ischemic stroke patients within 3–4.5 h after the stroke onset and under clinical observation as an effective and tolerable measure for the functional recovery after stroke.

## Data Availability Statement

The datasets generated for this study are available on request to the corresponding author.

## Ethics Statement

The studies involving human participants were reviewed and approved by Institute Review Board of National Taiwan University Hospital. The patients/participants provided their written informed consent to participate in this study.

## Author Contributions

Y-WC, S-FS, C-HC, S-JY, J-SJ, and L-ML contributed conception and design of the study. S-JY, J-SJ, and L-ML organized the database. J-SJ and L-ML performed the statistical analysis. Y-WC wrote the first draft of the manuscript. All authors contributed to acquisition, analysis or interpretation of patients' data, critical manuscript revision, read, and approved the submitted version. The corresponding author L-ML takes primary responsibility for communication with the journal and editorial office during the submission process, throughout peer review and during publication. The corresponding author is also responsible for ensuring that the submission adheres to all journal requirements including, but not exclusive to, details of authorship, study ethics and ethics approval, clinical trial registration documents, and conflict of interest declaration. The corresponding author should also be available post-publication to respond to any queries or critiques.

### Conflict of Interest

The authors declare that the research was conducted in the absence of any commercial or financial relationships that could be construed as a potential conflict of interest.
