# Supplementary material for: Intravenous Thrombolysis Administration 3–4.5 h After Acute Ischemic Stroke: A Retrospective, Multicenter Study
Source: Front Neurol. 2019 Oct 15;10:1038. doi: 10.3389/fneur.2019.01038 (PMC6803783; doi:10.3389/fneur.2019.01038)
Supplement: Supplementary file 1 [file Table_1.pdf]

Supplementary Table 1. Summary of stroke subtype based on TOAST classification and the percentage of large artery occlusion

Characteristics	Treatment (*N* = 374)	Control (*N* = 374)	*P*-value
**Ischemic stroke type**			
Large artery atherosclerosis	87 (23.3)	97 (25.9)	0.445
Small artery lacune	54 (14.4)	75 (20.1)	0.053
Cardioembolism	118 (31.6)	108 (28.9)	0.474
Other specific causes	11 (2.9)	18 (4.8)	0.235
Undetermined cause	103 (27.5)	75 (20.1)	0.020†
**Large artery occlusion**			
Cervical ICA	15 (4.3)	26 (7.4)	0.107
Intracranial ICA	12 (3.4)	13 (3.7)	0.842
MCA, M1	66 (18.8)	52 (14.8)	0.189
MCA, M2	18 (5.1)	11 (3.1)	0.255
VA	7 (2.0)	8 (2.3)	0.802
BA	7 (2.0)	16 (4.5)	0.088

Values are present as numbers (%).

P-value by chi-square test.

† Significant difference, P-value < 0.05.

TOAST, Trial of Org 10172 in Acute Stroke Treatment; ICA, internal carotid artery; MCA, middle cerebral artery; VA, vertebral artery; BA, basilar artery
