# Supplementary material for: Intravenous Thrombolysis Administration 3–4.5 h After Acute Ischemic Stroke: A Retrospective, Multicenter Study
Source: Front Neurol. 2019 Oct 15;10:1038. doi: 10.3389/fneur.2019.01038 (PMC6803783; doi:10.3389/fneur.2019.01038)
Supplement: Supplementary file 2 [file Table_2.pdf]

Supplementary Table 2. Medication before the onset of stroke

Characteristic	Treatment (*N*= 374)	Control (*N* = 374)	*P*-value
**Prestroke medication**			
Antiplatelets	82 (23.3)	99 (28.1)	0.168
Aspirin	63 (17.9)	89 (25.3)	0.022[†]
Clopidogrel	18 (5.1)	15 (4.3)	0.722
Others	12 (3.1)	4 (1.1)	0.074
Anticoagulants	10 (2.8)	16 (4.5)	0.318
Warfarin	9 (2.6)	13 (3.7)	0.517
NOACs	1 (0.3)	3 (0.9)	0.374
Anti-hypertensives	176 (50.0)	190 (54.0)	0.327
Anti-diabetic drugs	82 (23.3)	99 (28.1)	0.168
Lipid-lowering drugs			
Statins	43 (12.2)	54 (15.3)	0.274
Others	7 (2.0)	7 (2.0)	1.000
**No aforementioned medication**	**126 (32.8)**	**105 (29.8)**	**0.108**

Values are present as numbers (%).

† Significant difference, P-value < 0.05.

P- value by chi-square test

NOACs, non-vitamin K antagonistoral anticoagulants
