# Supplementary material for: Intravenous Thrombolysis Administration 3–4.5 h After Acute Ischemic Stroke: A Retrospective, Multicenter Study
Source: Front Neurol. 2019 Oct 15;10:1038. doi: 10.3389/fneur.2019.01038 (PMC6803783; doi:10.3389/fneur.2019.01038)
Supplement: Supplementary file 3 [file Table_3.pdf]

Supplementary Table 3. Comparison of Baseline Demographics Between Standard Dose and Low Dose Groups

**Characteristics**	**Standard dose (*N* = 182)**	**Low dose (*N* = 192)**	***P*-value**
Age (year)	62.5±13.1	69.5±12.4	<0.001[†]
Male, n (%)	123 (67.6)	128 (66.7)	0.938
Body weight (Kg)	66.5±14.3	64.4±11.5	0.125
NIHSS	10 (7, 17)	10 (6, 17)	0.227
Hypertension	136 (74.7)	146 (76.0)	0.861
Diabetes mellitus	62 (34.3)	75 (39.1)	0.371
Hyperlipidemia	101 (55.5)	98 (51.0)	0.448
Hypercholesterolemia	82 (45.1)	92 (47.9)	0.653
Hypertriglyceridemia	40 (22.0)	27 (14.1)	0.063
Cardiac disease	74 (40.7)	91 (47.4)	0.228
Atrial fibrillation	55 (30.2)	72 (37.5)	0.169
Ischemic heart disease	19 (10.4)	26 (13.5)	0.446
Valvular heart disease	9 (4.9)	6 (3.1)	0.527
Heart failure	16 (8.8)	14 (7.3)	0.732
Smoking			
Current smoker	51 (28.0)	52 (27.1)	0.931
Ex-smoker	7 (3.8)	19 (9.9)	0.036[†]
Previous ischemic stroke	32 (17.6)	43 (22.4)	0.302

Characteristics	Standard dose (*N* = 182)	Low dose (*N* = 192)	*P*-value
Previous transient ischemic attack	7 (3.8)	4 (2.1)	0.483
History of malignancy	9 (4.9)	12 (6.3)	0.747

Values are present as mean ± standard deviation, numbers (%), or median (interquartile range)

P-value by Student t-test or chi-square test.

[†]Significant difference, P-value < 0.05.

SD, standard deviation; NIHSS, National Institutes of Health Stroke Scale/score.
